# Salinity Modulates *Juncus acutus* L. Tolerance to Diesel Fuel Pollution

**DOI:** 10.3390/plants11060758

**Published:** 2022-03-12

**Authors:** Jesús Alberto Pérez-Romero, José-María Barcia-Piedras, Susana Redondo-Gómez, Isabel Caçador, Bernardo Duarte, Enrique Mateos-Naranjo

**Affiliations:** 1 Departamento de Biología, Instituto Universitario de Investigación Marina (INMAR), Universidad de Cádiz, 11510 Puerto Real, Spain; 2 Department of Ecological Production and Natural Resources Center IFAPA Las Torres, Tomejil Road Sevilla, Cazalla Km 12’2, 41200 Alcalá del Río, Spain; bapi@us.es; 3 Departamento de Biología Vegetal y Ecología, Facultad de Biología, Universidad de Sevilla, 1095, 41080 Sevilla, Spain; susana@us.es (S.R.-G.); emana@us.es (E.M.-N.); 4 MARE—Marine and Environmental Sciences Centre, Faculty of Sciences, University of Lisbon Campo Grande, 1749-016 Lisbon, Portugal; micacador@fc.ul.pt (I.C.); baduarte@fc.ul.pt (B.D.); 5 Departamento de Biologia Vegetal, Faculdade de Ciências da Universidade de Lisboa, Campo Grande, 1749-016 Lisbon, Portugal

**Keywords:** petroleum-derived substances (PDS), chlorophyll a fluorescence, halophyte, abiotic stress, physiology

## Abstract

Soil contamination with petroleum-derived substances such as diesel fuel has become a major environmental threat. Phytoremediation is one of the most studied ecofriendly low-cost solutions nowadays and halophytes species has been proved to have potential as bio-tools for this purpose. The extent to which salinity influences diesel tolerance in halophytes requires investigation. A greenhouse experiment was designed to assess the effect of NaCl supply (0 and 85 mM NaCl) on the growth and photosynthetic physiology of *Juncus acutus* plants exposed to 0, 1 and 2.5% diesel fuel. Relative growth rate, water content and chlorophyll a derived parameters were measured in plants exposed to the different NaCl and diesel fuel combinations. Our results indicated that NaCl supplementation worsened the effects of diesel toxicity on growth, as diesel fuel at 2.5% reduced relative growth rate by 25% in the absence of NaCl but 80% in plants treated with NaCl. Nevertheless, this species grown at 0 mM NaCl showed a high tolerance to diesel fuel soil presence in RGR but also in chlorophyll fluorescence parameters that did not significantly decrease at 1% diesel fuel concentration in absence of NaCl. Therefore, this study remarked on the importance of knowing the tolerance threshold to abiotic factors in order to determine the bioremediation capacity of a species for a specific soil or area. In addition, it showed that NaCl presence even in halophytes does not always have a positive effect on plant physiology and it depends on the pollutant nature.

## 1. Introduction

Petroleum-derived substances (PDSs) such as diesel, have become a common soil pollutant [1]. Nowadays it is considered a global problem because of the harmful effects that these compounds exert on the natural environment [1]. Contamination with PDSs can cause phytotoxicity in several plant species [2]. Between these PDSs, diesel fuel is a petroleum product widely used nowadays [3]. This substance is a complex mixture of petroleum hydrocarbons containing several compounds from alkanes to naphthalenes [4]. Soils polluted with diesel fuel can negatively affect the physiology [5], development [4], germination, and growth of plant species [6,7]. This phytotoxicity is affected by many variables such as the time that has passed since the contamination [6,8], the contamination degree [7,9] and the plant species [9,10]. In addition, soil contamination with PDSs constitutes a potential danger to human health and life [1]. Therefore, efforts have been undertaken in order to find effective, economically affordable and environmentally friendly methods to restore contaminated soils [11,12]. One of these methods that could be implemented in contaminated soils is bioremediation, which is a technique that is widely discussed to deal with heavy metal pollution [4,13,14]. Nevertheless, it could be useful to cope with PDS contamination as an environmentally friendly method [11].

The use of plants to restore contaminated soils and waters is under active investigation [4,14]. Plants provide an optimal environment for microbial proliferation in the rhizosphere [15]. As a result, plants seem to encourage organic contaminant reduction [16,17]. However, plants are very sensitive and respond rapidly to diesel presence [18]. Therefore, it is important to identify plants capable of growing in diesel-contaminated soils [19]. *Juncus* species have been proposed as a bio-tool for wetland restoration projects around the world [20,21] due to their ability to cope with heavy metals [22,23,24] and petroleum-derived substances such as diesel [25]. A recent study developed in *J. acutus* identified that its capacity to bioremediate heavy metal contaminated soils is modulated by other environmental factors as soil salinity [26] or even by the contamination existing in the original population of the seeds used [14]. Hence, knowledge of the extent to which salinity might modulate the physiological responses of *J. acutus* to diesel fuel concentration is necessary for a realistic assessment of its toxicity thresholds and its potential for the remediation of diesel-polluted saltmarshes. This study employed a factorial experiment which aimed to: (1) investigate the influence of NaCl on the growth responses of *J. acutus* plants exposed to different diesel fuel concentrations and (2) determine the extent to which this influence could be accounted for by impacts on its photosynthetic apparatus. Accomplishing these two main objectives will help us to obtain a more realistic vision of *J. acutus* or other halophytes potential as bio-tools knowing how their resistance to PDS could be modified under salinity conditions representative of their natural environment.

## 2. Results

### 2.1. Effects of Diesel and NaCl on Relative Growth Rate and Relative Water Content

**Figure 1:**
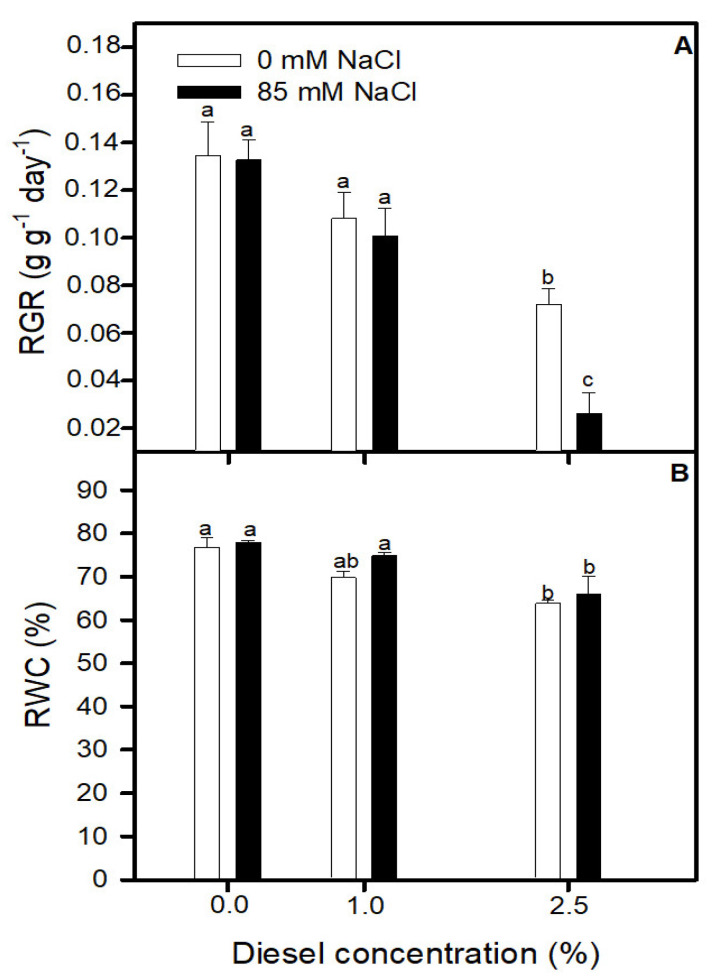
Relative growth rate (RGR) (**A**), and total relative water content (WC) (**B**) of *Juncus acutus* in response to treatment with a range of diesel concentrations at 0 and 85.5 mM NaCl for 20 d. Values represent mean ± SE, *n* = 10. Different letters indicate means that are significantly different from each other (LSD test, *p* < 0.05).

After 20 days of experimental conditions, diesel concentration showed a significant deleterious effect on *J. acutus* growth as indicated RGR values (GLM_RGR_ Diesel *p* < 0.01 F = 18.54). In addition, the interaction between diesel and NaCl also showed a significant effect (GLM_RGR_ Diesel×NaCl *p* < 0.05 F = 4.98). As it is shown in Figure 1A, RGR decreased with the increment of diesel percentage in the growth solution. Being significant, the differences between the plants grown at 0% and 2.5% treatments (LSD test *p* < 0.05). Nevertheless, differences between salinity treatments at the same diesel levels were found only at 2.5% where plants grown at 85 mM NaCl showed lower values than plants grown at 0 mM NaCl (LSD test *p* < 0.05). WC showed a similar trend but without significant differences between salinities concentrations treatments (GLM_WC_ NaCl *p* > 0.05 F = 1.0, GLM_WC_ Diesel×NaCl *p* > 0.05 F = 0.02) (Figure 1B).

### 2.2. Effects of Diesel and NaCl on Chlorophyll a Fluorescence

**Figure 2:**
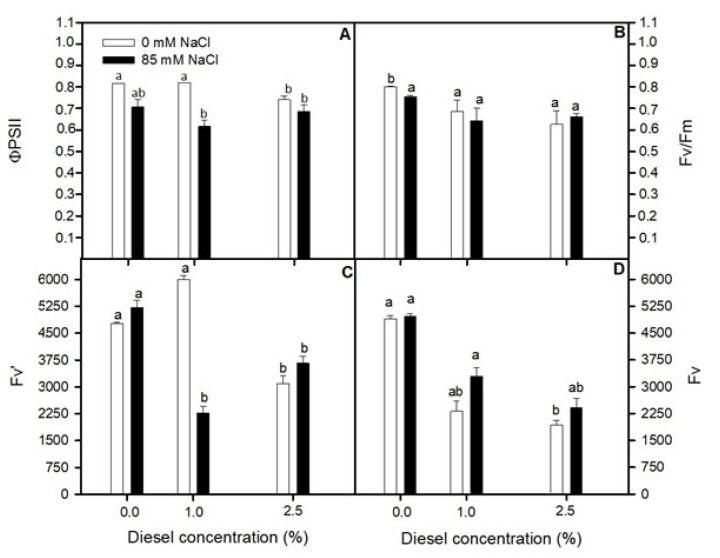
Maximum quantum efficiency of PSII photochemistry, F*v*/F*m* (**A**), Light and dark-adapted quantum yield of primary photochemistry, equal to the efficiency by which a PS II trapped photon will reduce QA to QA, ΦPSII (**B**), variable fluorescence (F*v*) (**C**) and relative variable fluorescence (F*v*′) (**D**) in dark-adapted randomly selected, primary branches of *Juncus acutus* in response to treatment with a range of diesel concentrations at 0 and 85.5 mM NaCl for 20 d. Values represent mean ± SE, *n* = 7. Different letters indicate means that are significantly different from each other (LSD, *p* < 0.05).

Operational quantum yield (F_v_/F_m_) decreased for plants grown at 85 mM NaCl when the diesel was present at the grown solution (Figure 2A). However, this trend was not significant (GLM _F_v_/F_m__ Diesel×NaCl *p* > 0.05 F = 0.02). In addition, F_v_/F_m_ values at 85 mM NaCl independently of diesel concentration were significantly lower than at control condition (0mM NaCl and 0% diesel) (LSD test *p* < 0.05). Maximum quantum yield (Φ_PSII_) showed significantly lower values at 85 mM NaCl for all the diesel treatments (Figure 2B) (GLM_ΦPSI_ NaCl *p* < 0.05 F = 6.18). Nevertheless, Φ_PSII_ did not decrease compared to control conditions when growing at 1% diesel without NaCl (LSD test *p* > 0.05). Maximum and operational variable fluorescence (F_v_ and F′_v_, respectively) (Figure 2C,D) overall decreased with the presence of diesel in the grown solution (GLM_F_v__ Diesel *p* < 0.01 F = 10.00, GLM_F_v_′_ Diesel *p* < 0.01 F = 25.84). At 0% and 2.5% diesel concentrations, there were no significant differences between plants grown at different salinities for both parameters (LSD test *p* > 0.05). However, at 1% diesel concentration F_v_′ was higher at 0 mM NaCl than at 85 mM NaCl although it showed no significance (LSD test *p* > 0.05).

### 2.3. Effects of Diesel and NaCl on Kautsky Curves Parameters

**Figure 3:**
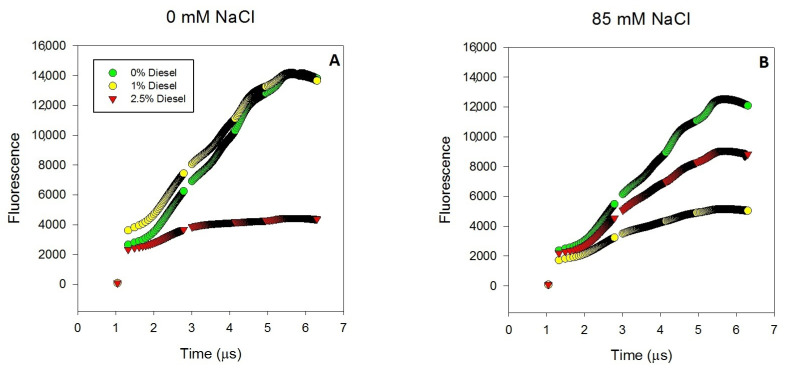
Transient fluorescence (Kautsky curves) of dark-adapted leaves of *Juncus acutus* in response to treatment with a range of diesel concentrations at 0 mM NaCl (**A**) and 85.5 mM NaCl (**B**) for 20 d. Values represent the mean of five measurements per treatment combination.

**Figure 4:**
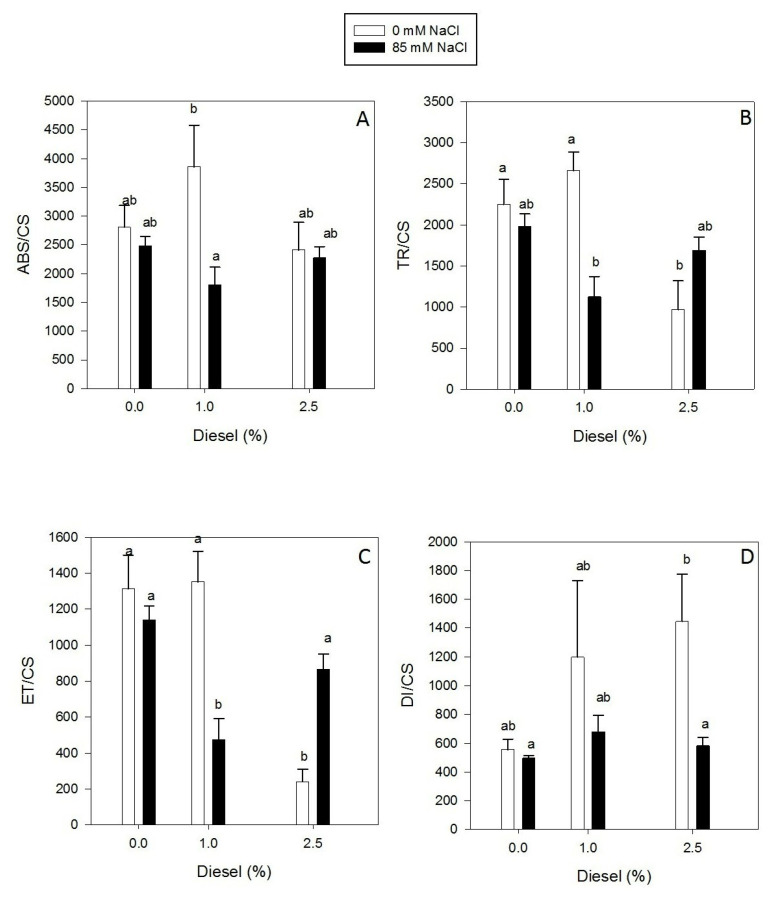
Absorbed energy flux, ABS/RC (**A**), trapped energy flux, TR/RC (**B**) electron transport energy flux, ET/RC (**C**), dissipated energy fluxes, DI/RC (**D**) per reaction center in dark-adapted randomly selected, primary branches of *Juncus acutus* in response to treatment with a range of diesel concentrations at 0 and 85.5 mM NaCl for 20 d. Values represent mean ± SE, *n* = 5. Different letters indicate means that are significantly different from each other (LSD, *p* < 0.05).

The OJIP curve (Figure 3) showed similar variable fluorescence values for plants grown at 0 and 85 mM NaCl at 0% of diesel. The maximum fluorescence reached had a decrease of almost 65% at 1% of diesel when the NaCl was added to the grown solution. In plants grown at 2.5% of diesel, the decrease was seen at both salinities being more accused at 0 mM NaCl than at 85 mM NaCl. ABS/CS values did not show differences with the diesel concentration at both salinities (Figure 4A) (GLM_ABS/CS_ *p* > 0.05 F = 1.945076). However, the absorbed energy flux (ABS/CS) showed values significantly higher for plants grown at 0 mM NaCl than for those grown at 85 mM NaCl at 1% diesel (LSD test *p* < 0.05). Trapped (TR/CS) and transported (ET/CS) energy fluxes followed a similar trend for plants grown at 1% diesel (Figure 4B,C). In addition, in both parameters a significant effect of both NaCl and diesel were found (GLM_TR/CS_ NaCl *p* < 0.05 F = 4.86 Diesel *p* < 0.01 F = 10.17, GLM_ET/CS_ NaCl *p* < 0.05 F = 6.36 Diesel *p* < 0.01 F = 22.19). Moreover, both of them were higher for plants grown at 85 mM NaCl than for plants grown in the absence of NaCl at the highest diesel concentration (LSD test *p* < 0.05). Dissipated energy flux (DI/CS) increased with diesel concentration significantly in plants grown at 0 mM NaCl reaching its maximum values at 2.5% diesel (Figure 4D) (GLM_DI/CS_ Diesel *p* < 0.01 F = 8.87). At 85 mM NaCl, it remained at similar values for all diesel concentrations tested and showed lower values than in the absence of NaCl overall (LSD test *p* > 0.05).

**Figure 5:**
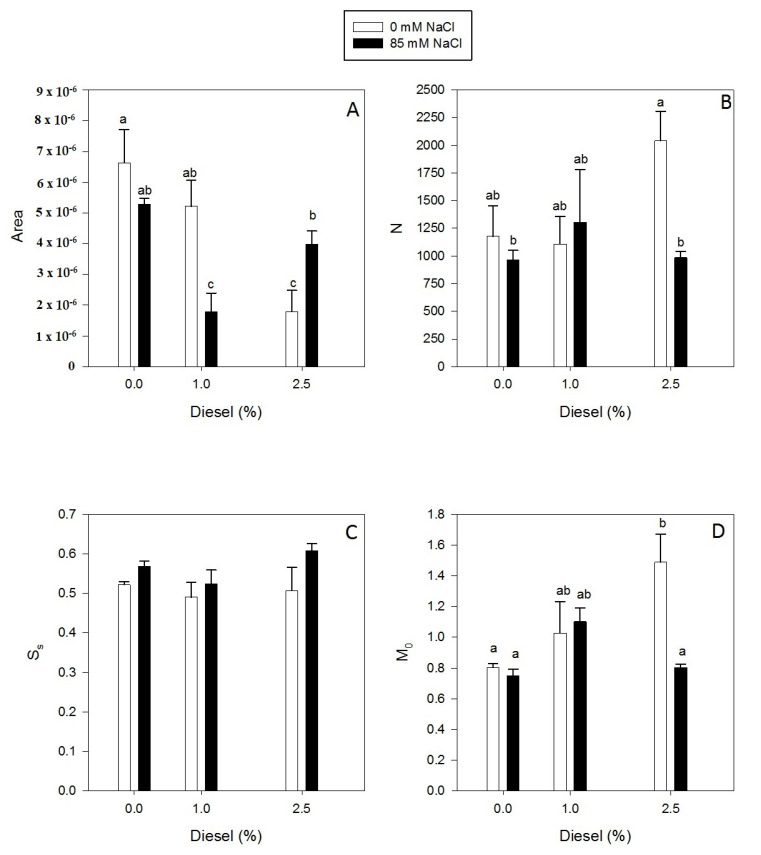
Oxidized quinone pool size available for reduction, Area (**A**), Reaction centre turnover rate, N (**B**), relative pool size of plastoquinone, S_S_ (**C**) and net rate of PS II RC closure, M_0_ (**D**) in dark-adapted randomly selected, primary branches of *Juncus acutus* in response to treatment with a range of diesel concentrations at 0 and 85.5 mM NaCl for 20 d. Values represent mean ± SE, *n* = 5. Different letters indicate means that are significantly different from each other (LSD, *p* < 0.05).

**Figure 6:**
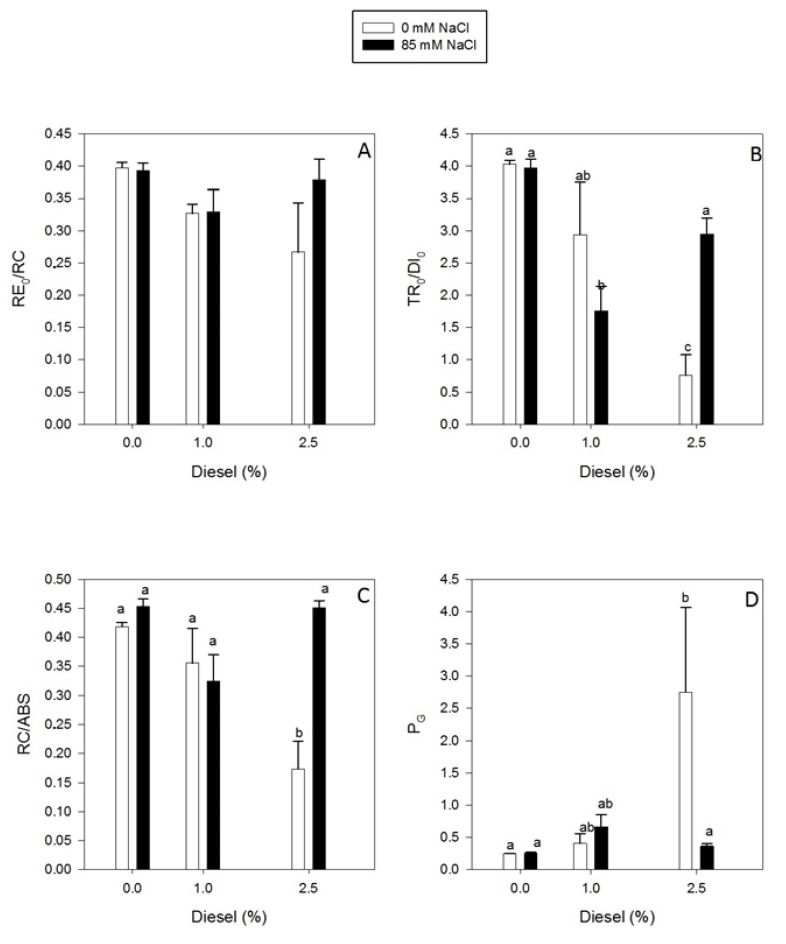
Electron transport flux until PSI acceptors per PSII, RE_0_/RC (**A**), flux ratio trapping per dissipation;, TR_0_/DI_0_ (**B**), density of reaction centres on chlorophyll *a* basis, RC/ABS (**C**) and grouping probability, P_G_ (**D**) per reaction center in dark-adapted randomly selected, primary branches of *Juncus acutus* in response to treatment with a range of diesel concentrations at 0 and 85.5 mM NaCl for 20 d. Values represent mean ± SE, *n* = 5. Different letters indicate means that are significantly different from each other (LSD, *p* < 0.05).

Single turnover (S_S_) and RE_0_/RC (Figure 5A and Figure 6A) did not show significant differences for treatments applied (GLM_Ss_ *p* > 0.05 F = 2.41, GLM_RE0/RC_ *p* > 0.05 F = 2.49). However, N, M_0_ and P_G_ (Figure 5B,C and Figure 6D) values increased with diesel concentration for plants grown at 0 mM NaCl (GLM_N_ Diesel *p* < 0.01 F = 6.07, GLM_Mo_ Diesel *p* < 0.001 F = 18.42, GLM_PG_ Diesel *p* < 0.01 F = 12.27). Under 85 mM NaCl these variables shown no evident changes independently of the diesel concentration applied. Contrarily, Area, TR_0_/DI_0_ and RC/ABS (Figure 5A and Figure 6B,C) decreased with diesel concentration at 0 mM NaCl (GLM_Area_ Diesel *p* < 0.001 F = 17.39, GLM_TR0/DI0_ Diesel *p* < 0.001 F = 25.83, GLM_RC/ABS_ Diesel *p* < 0.001 F = 20.39). In addition, all of them were affected by the interaction between NaCl and diesel presence (GLM_Area_ Diesel×NaCl *p* < 0.05 F = 6.24, GLM_TR0/DI0_ Diesel×NaCl *p* < 0.01 F = 8.08, GLM_RC/ABS_ Diesel×NaCl *p* < 0.01 F = 12.41). In addition, these values reached their minimum at 85 mM NaCl and 1% diesel.

**Figure 7:**
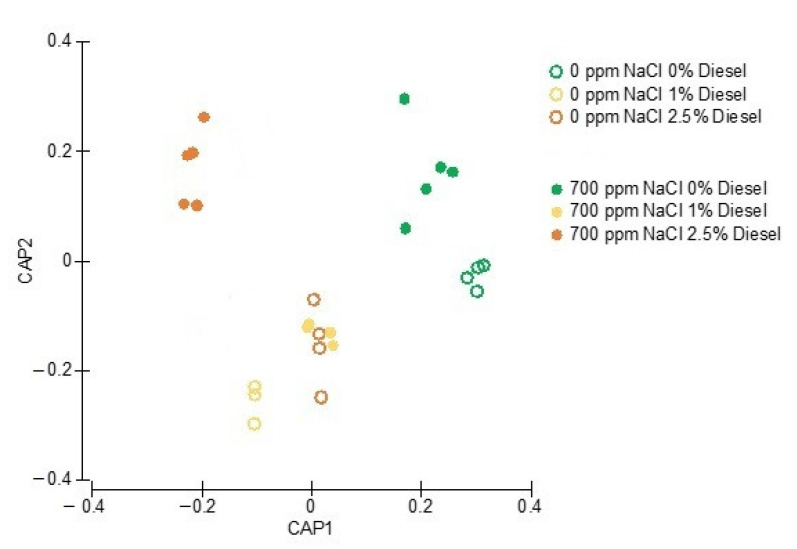
Canonical Analysis of Principal (CAP) components based in the Kautsky curve fluorescence data points of the *J. acutus* samples exposed to diesel with (empty circles) and without (filled circles) NaCl supplementation.

In analyzing the full Kautsky fluorescence profile, it is possible to evidence that the different diesel and salt treatments induced differential photochemical responses. (Figure 7). Samples exposed in the absence of diesel and salt appear to have a high degree of similarity in photochemical terms being projected in the same CAP sector. In the absence of external NaCl application, there is an overlap of the samples exposed to intermediate concentrations of diesel indicating that the 1% and 2.5% concentrations lead to similar impacts at the photochemical level.

**Figure 8:**
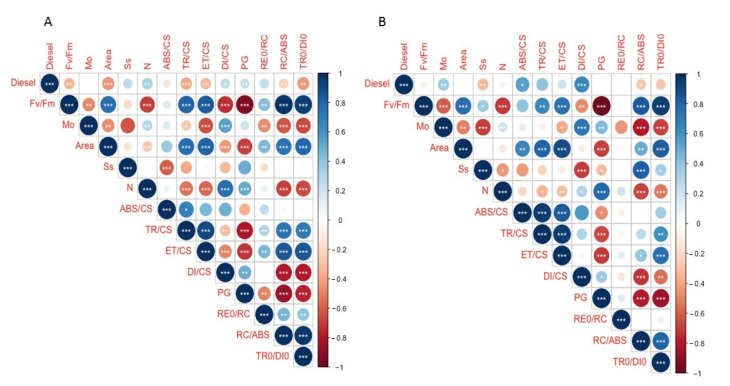
Spearman correlations coefficients (r^2^) between the evaluated photochemical variables and the diesel concentrations applied in plants grown without (**A**) and with (**B**) NaCl supplementation.

It was also possible to assess the relationship of the photochemical variables attained from the OJIP test and the exogenous diesel concentration applied in the absence (Figure 8A) and in the presence (Figure 8B) of NaCl. Without salt application, several photochemical traits such as the size of the oxidized quinone pool and the absorbed, trapped and transported energy fluxes showed significant inverse correlations with the diesel concentration applied. Upon the joint application of diesel and salt, all these traits exhibited an inverse trend, as well as M_0_.

## 3. Discussion

The results here obtained for growth are in concordance with previous studies for diesel or PDSs in different plant species [1,4,6,9,27,28]. Thus, we found that in absence of salinity *J. acutus* growth was never reduced more than 50% under polluted conditions compared with control conditions (20% and 47% for diesel concentration of 1% and 2.5%, respectively). This observed decrease was inferior to the one verified for other halophytes such as *Spartina argentinensis* that presented a growth reduction of about 65% upon the application of 1% diesel [4]. These results indicate that *J. accutus* might be a diesel-tolerant plant. However, a reduction in plants grown at 85 mM NaCl was greater than at 0 mM NaCl. This decrease was around 80% at 2.5% diesel concentration. This response could be related to the fact that *Juncus* species subjected to salt in the grown solution trend to reduce their growth in order to activate some defense mechanisms [29]. Pawluśkiewicz et al. [19] found that diesel induces a defense mechanism in which plants extend their cells in order to increase the radicle surface and develop new root hairs due to oil causing clogging of the vascular cells, used for water intake. A reduction in growth induced by the higher NaCl concentration tested could lead to an inhibition of the root development. This reduction could be led by diesel stress and could cause the greater RGR decrease seen in plants grown at 2.5% diesel concentration and 85 mM NaCl.

The reduction in growth could also be related to a decrease in *J. acutus* photosynthetic capacity as indicated in previous studies developed in different species that showed a marked reduction in the net photosynthetic rate of plants exposed to diesel or PDSs [4,30]. In the present work, no signs of chlorosis could be observed in plants grown under non-saline conditions and in the presence of diesel. This is in contrast to the observed by Redondo-Gómez et al. [4] for the halophyte *S. argentinensis* when subjected to 1%, 2% and 3% diesel fuel concentration. The lack of chlorosis could be related to the absence of negative effects of the diesel fuel against chlorophyll a and carotenoid concentration as Redondo-Gómez et al. [4] has reported. In fact, there is a marked relationship between the maintaining of pigments antenna complexes and the efficiency of photosynthetic apparatus [31]. Thus, our results showed that although there was a negative effect in the photosystems photochemical apparatus efficiency of *J. acutus* under diesel exposition, this effect was amplified by NaCl presence.

Thereby, the operational variable fluorescence (F_v_) did not show this negative trend caused by the salinity. In fact, a similar decrease was observed in the presence of diesel regardless of the salinity tested. Nonetheless, F_v_’ only decreased at the highest diesel concentration, showing the most negative effect at 85 mM NaCl. Similarly, these trends were reflected in the response of Photosystem II quantum yield (F_v_/F_m_), which showed a decrease with the increment of diesel concentration in the soil, being this decrease less notorious in the absence of NaCl. This response pattern was also observed in OJIP derived parameters. Thus, ABS/RC and ET/RC values variations indicated that there was energy absorbed by the PS II that was not transferred to the electron transport chain mainly in plants grown at 0 mM NaCl and 2.5% diesel, which could be linked with possible damage on the photosystems. Moreover, the observed OJIP curve shape variation between each of the specific diesel and salinity combination treatments could be related to the plant’s ability to dissipate energy excess as heat, accordingly with recorded dissipated energy flux per leaf cross-section (DI/CS) pattern. In fact, DI/CS parameter is known to be indicative of anti-stress mechanisms through energy dissipation. Duarte et al. [32] related it with strategies against oxidative stress such asas NPQ, xantophyll cycle or photorespiration. *J. acutus* plants that were grown at 85 mM NaCl and diesel presence did not show an increment in DI/CS although their ABS/CS and TR/CS compared with their ET/CS indicated that there was energy lost. This response would indicate that salinity excess contributed to the decline of the reaction centers available for photon harvesting in response to diesel concentration.

All these significant changes in the photochemical traits were also evidenced when using the whole photochemical process. The clear separation of the test groups indicated especially at the two highest diesel concentrations; highlighting the potential of using photochemical tools to address the toxicity effects in *J. acutus*. In fact, previous studies reinforced the potential of this plant as a bio-monitor of contaminants using the JIP-test as a basis [33], and thus this application of photochemical tools for biomonitoring of diesel contamination can be of added value in order to monitor non-invasively the phytoremediation process.

Mateos-Naranjo et al. [26] described an ameliorative effect of NaCl at 85 mM for *J. acutus* when it was exposed to Zn. In their essay, NaCl presence improved the PS state and increased the Zn threshold tolerance of this species. They found that NaCl presence in the grown solution reduced the Zn uptake and translocation reducing the amount of Zn accumulated in *J. accutus* tissues [26]. In this case, salinity in the grown solution seemed to increase the negative effects of diesel in *J. acutus.* This could be due to the effect of diesel on soil quality [34]. Martina et al. [34] found for two species (*Lupinus sativa* and *Raphanus sativus*) that the reduction in growth and dry mass due to PDS presence was more related to the effect of PDS over nutrient availability and less related to the accumulation of these products in plant tissues. Diesel in soil could work as a physical barrier limiting the water and nutrients intake and therefore reducing the growth capacity and physiological performance of plant species [35,36]. Compounds as complex as diesel are hard to absorb by plants for the growth solution, only low molecular weight or simple structure compounds are easily acquired by roots [37]. Hence, it is hard for diesel or PDS to accumulate in plant tissues even if there is a high concentration of them in the soil as Martina et al. [34] have shown. Salinity-derived stress is usually related to water stress [38] reducing plant growth and affecting photosynthesis fitness [39]. Thus, as NaCl also contributed to reducing the availability of water for plants, both diesel and NaCl negative effect synergized and produced the results seen in our experiment differing from what Mateos-Naranjo et al. [26] found due to differences between Zn induced stress and PDS induced stress mechanisms.

## 4. Material and Methods

### 4.1. Plant Material

*Juncus acutus* were grown from seeds collected from different individuals (*n* = 20) randomly selected from a stable population in Doñana National Park (Huelva, SW Spain). The seeds were transported to a germination chamber (ASL Aparatos Científicos M-92004, Madrid, Spain) under the following conditions: photoperiod, 16/8 h light/darkness; temperature, 24/15 °C; photon flux rat (400–700 nm), 35 μmol m^−2^ s^−1^. Seeds recently germinated were transferred to individual plastic pots (12 cm in depth, 0.5 L total volume) filled with sand and placed in a glasshouse (University of Seville, Greenhouse Service) at a controlled temperature of 25 ± 3 °C, and a relative humidity of 40–60%, with natural daylight (maximum quantum flux rate of 1000 μmol m^−2^ s^−1^). Pots were irrigated with nutrient solution [40] and kept under the abovementioned conditions for three months before the onset of the experimental treatments.

### 4.2. Diesel and NaCl Experimental Stress Treatments

When *J. acutus* seedlings were developed, pots were randomly assigned to three diesel treatments 0%, 1% and 2.5% (concentrations of 0 L Kg^−1^, 1.2 × 10^−2^ L Kg^−1^ and 3 × 10^−2^ L Kg^−1^) in factorial combination with two NaCl concentrations (0 and 85 mM) for 20 days. This salinity concentration was chosen due to being representative of the natural environment where these species inhabit [14]. NaCl concentrations were established by combining Hoagland’s solution with appropriate amounts of NaCl. Therefore, at the beginning of the experiment, the pots were placed in plastic trays containing appropriate solutions to a depth of 1 cm (10 replicate pots per stress treatment combination). Diesel concentrations were established with a mix of diesel and sand in the proportion before mentioned. Sand and diesel were mixed in plastic bags, and these were kept closed for 24 h before filling the pots with the mixture. In order to avoid changes in NaCl concentration caused by water evaporation from the nutrient solution, levels in the trays were monitored continuously throughout the experiment and topped up to the marked level with Hoagland’s solution (without additional diesel or NaCl). Furthermore, the pH of the solution was monitored and adjusted to 6.5–7.0. The entire solution in the trays was renewed weekly and their positions were changed randomly every 2 days to avoid effects of environmental heterogeneity inside the glasshouse. After 20 days of exposure to these described treatments, measurements of growth and chlorophyll fluorescence were carried out.

### 4.3. Growth Measurements and Water Content

At the beginning of the experiment, four plants from each treatment were harvested. At the of 20 days, ten more plants for each treatment were harvested. Plants were divided into roots and shoots and these biomass fractions were oven-dried (60 °C for 48 h) and then weighed. The relative growth rate (RGR) of whole plants was calculated using the formula:

RGR = (ln B_f_ − ln B_i_) × D^−1^ (g g^−1^ day^−1^) (1)

where B_f_ = final dry mass, B_i_ = initial dry mass (the mean of the four plants from each treatment sampled at the beginning of the experiment) and D = duration of the experiment (days).

Moreover, the fresh weight of these ten plants was also assessed and the relative water content was calculated as:

WC = (FW − DW) × FW^−1^ × 100 (2)

where FW = fresh weight of the sample after collecting it and DW = dry weight of the sample after being oven-dried.

### 4.4. Chlorophyll Fluorescence Measurement

Modulated chlorophyll fluorescence measurements were performed on randomly selected fully developed photosynthetic tillers using a FluorPen FP100 (Photo System Instruments, Drásov, Czech Republic) on light and 30 min dark-adapted tillers after 20 days of treatment (*n* = 7, per treatment). As Schreiber et al. [41] described, light energy yields of photosystem II (PS II) reaction centres were determined with a saturation pulse method. Maximum fluorescence signal across time was estimated by using a saturating light pulse of 0.8 s with an intensity of 8000 μmol m^−2^ s^−1^. Minimum fluorescence (F′_0_), maximum fluorescence (F′_m_) and operational photochemical efficiency values of light and dark-adapted leaves were compared. Quantum yield of PS II (QY) and relative Quantum yield of PS II (Q’Y) were calculated as F_v_/F_m_ and Φ_PSII_ respectively. In dark-adapted leaves, Kautsky curves, or JIP-test, were also measured (*n* = 5, per treatment) following the Duarte et al. [42] method. Pre-programmed OJIP protocols of the FluorPen were used for this purpose. All derived parameters for OJIP were calculated according to Strasser et al. [31].

### 4.5. Statistical Analysis

Statistical software package R 4.0.2 was used to perform the entire statistic. Generalized linear models (GLM) were used to analyze the interactive effects of diesel and NaCl concentrations (as categorical factors) on the growth and physiological parameters (as dependent variables) of *J. acutus* plants. Multiple comparisons were analyzed by an LSD (post hoc) test. Before statistical analysis, Kolmogorov-Smirnov and Brown-Forsythe tests were used to verify the assumptions of normality and homogeneity of variances, respectively. Primer 6 software [43] was used to carry out multivariate statistical analyses using non-parametric multivariate analysis packages. After data normalization, the resemblance matrix of all variables (based on Euclidean distances) was analyzed using Canonical Analysis of Principal Coordinates (CAP). This methodology was employed to generate statistical multivariate models based on the bio-optical data and to classify and separate the different salt and diesel treatments, as well as to test the sensitivity of these traits as descriptors of the plant response to the tested treatment. This multivariate approach is insensitive to heterogeneous data and frequently used to compare different sample groups using the intrinsic characteristics (elemental concentrations) of each group [31,44]. Spearman correlations were performed using corrplot package in R-Studio.

## 5. Conclusions

These results highlight the importance of characterizing the environment where plant species are growing to assert the ability of a potential bio-tool or to determine the tolerance threshold of a species. *Juncus acutus* showed great tolerance to diesel fuel concentration on the grown solution with a decrease in its RGR being less than 25% at 2.5% diesel concentration in 0 mM NaCl conditions. However, NaCl concentration of 85 mM decreased this tolerance reducing the RGR by more than 80% for the same diesel fuel concentration. This was related to damage in the photosystem electron transport capacity as the OJIP derived parameters demonstrated. This result displayed the importance of the study threshold tolerance of species taking into account other environmental factors that could occur on their distribution. Showing that these other factors, such as NaCl salinity in this example, could drastically change the viability of a species as a phytoremediation tool. Hence, further studies must be conducted as plants using phytoremediation are going to be interacting with a complex environment. In these environments, plant species are going to be exposed to different abiotic factors that may reduce their capacity to cope with contaminants and their utility as bio-tools. Field research and more in-depth essays would be useful to advance in this concern.

## Data Availability

Data is contained within the article.
